# Bibliometric evaluation of 2011–2021 publications on hydrogen sulfide in heart preservation research

**DOI:** 10.3389/fcvm.2022.941374

**Published:** 2023-01-09

**Authors:** Mingcong Chen, Qian Zhou, Fei Wu, Fang Sun, Yang Meng, Yang Zhang, Mingyi Zhao

**Affiliations:** Department of Pediatrics, The Third Xiangya Hospital, Central South University, Changsha, Hunan, China

**Keywords:** heart preservation, bibliomeric analysis, CiteSpace, visualization analysis, hydrogen sulfide

## Abstract

**Background:**

Hydrogen sulfide (H_2_S) is known for its unpleasant odor and severe toxicity. However, an in-depth study of H_2_S showed that it can be used as an important messenger, which can play important physiological and pathological roles *in vitro* and *in vivo*. In recent years, the application of H_2_S in the field of cardiac preservation has attracted the interest and attention of scholars worldwide. H_2_S plays an effective and protective role in cardiac ischemia/reperfusion injury through antioxidant, anti-inflammatory, and antiapoptotic mechanisms.

**Objective:**

The purpose of this study is to analyze the current scientific achievements on the application of H_2_S in the field of cardiac preservation and to provide new ideas for further research.

**Methods:**

TS = (“hydrogen sulfide” OR “hydrogen sulfide”) AND TS = (“cardiac” OR “heart” OR “myocardium” OR “hearts”) AND TS = (“reperfusion” or “transplantation” or “implanted” or “transplant” or “implantation” or “migration” or “preservation” or “grafting” OR “ischemia” OR “perfusion” or “conservation” or “preserve” or “reservation”) AND DT = (Article OR Review) AND LA = (English) were used as search strategies for data collection from the Science Citation Index-Expanded database of the Web of Science Core Collection. CiteSpace 5.8. R3 and Microsoft Office Excel 2019 were used for data analysis.

**Results:**

A total of 429 related articles were included, and the total number of articles showed a fluctuating upward trend. We used CiteSpace 5.8. R3 and Microsoft Excel 2019 to evaluate and visualize the results, analyzing institutions, countries, journals, authors, co-cited references, and keywords.

**Conclusions:**

As increasing evidence shows that H_2_S plays an indispensable role in the field of cardiac preservation, its mechanistic research and clinical application may become the main focus of future research.

## 1. Introduction

For centuries, hydrogen sulfide (H_2_S) has been known for its unpleasant odor and severe toxicity. However, with the in-depth study of H_2_S, it has been found that as an endogenous gas-signaling molecule, such as nitric oxide (NO) and carbon monoxide (CO), it can be produced in the human body and can be involved in physiological and pathological processes in multiple systems (1, 2). For example, it plays an important role in the central nervous (3), cardiovascular (4), renal (5), reproductive (6), respiratory (7), and digestive systems. Several enzymes, mainly cystathionine β-synthase (CBS) (8), cystathionine γ-lyase (CSE) (9, 10), and 3-mercaptopyruvate sulfurtransferase (MST) (11, 12), precisely and strictly regulate the production of H_2_S in the body. Among them, CBS is the main H_2_S synthase in the central nervous system (13); in contrast, CSE and MST are more widely distributed in mammalian tissues, especially in the cardiovascular system (14, 15).

Heart transplantation is the standard care for patients with end-stage heart failure (HF). Heart transplantation is limited by the inability to safely preserve the donor heart long-term (16). Myocardial performance and long-term prognosis after transplantation mainly depend on ischemia time, that is, the level of ischemia/reperfusion injury (I/R) (17, 18). Therefore, it is necessary to find new-organ preservation media that reduces reperfusion injury during cardiac ischemia time to maintain the viability of the heart as long as possible. As an important gas transmitter, H_2_S can play an effective protective role in the heart undergoing I/R *in vitro* and *in vivo* through antioxidative (19), anti-inflammatory, and antiapoptotic mechanisms (20).

Exogenous H_2_S in the form of inorganic sulfide salts, such as sodium hydrosulfide (NaHS), has been developed. Hu et al. confirmed that Krebs–Henseleit (KH) solution with NaHS as a preservation solution had better protection for isolated rat hearts than KH solution without NaHS, which was manifested in a shorter fibrillation time and recovery time of effective contraction after reperfusion, indicating that H_2_S has a protective effect on the myocardium after reperfusion (21). However, the difficulty in providing a stable and controllable H_2_S preservation solution is an important factor that limits the application of H_2_S in cardiac preservation. Sun et al. demonstrated that a novel long-term and slow-releasing H_2_S system, namely DATS-MSN, could inhibit the Toll-like receptor 4/Nuclear transcription factor-κB (TLR4/NF-κB) pathway and NOD-, LRR-, and pyrin domain-containing protein 3 (NLRP3) activation to exert anti-inflammatory effects, thereby exerting effective cardio-protection and prognosis improvement (22). This slow-releasing H_2_S donor provides more durable and stable H_2_S release than inorganic sulfide salts, reflecting its optimistic application prospects. However, there is still a lack of in-depth and comprehensive analysis and research on the application of H_2_S in heart preservation. Bibliometrics adopts quantitative research methods, including mathematics and statistics, to quantitatively analyze the literature and measure its influence to help researchers understand related fields more quickly (23). In addition, bibliometrics also identifies frontier hotspots and future research directions.

To date, there is no bibliometrics on the application of H_2_S in heart preservation. Based on CiteSpace V5.8. R3 and Microsoft Office Excel 2019, this study conducted a visual analysis of 429 related studies, such as research trends, national/regional cooperation, institutional cooperation, author citation, and keyword co-occurrence. It effectively and intuitively analyses the research trends of the past decade and predicts future research hotspots. Moreover, it can further guide scholars in this field to conduct more in-depth and comprehensive research on H_2_S in heart preservation.

## 2. Methods

### 2.1. Search strategies

Data were downloaded from the Science Citation Index-Expanded database of the Web of Science Core Collection (WoSCC) on a single day, 10 March 2022. In the process of data retrieval, the accuracy and comprehensiveness of the original data are crucial, and the retrieval strategy needs to be adjusted and optimized constantly. We selected relevant literature in the past 10 years (2011–2021) to study the current status and trend of development over the past 10 years. The search terms used were as follows: (“hydrogen sulfide” OR “hydrogen sulfide”) AND (“cardiac” OR “heart” OR “myocardium” OR “hearts”) AND (“reperfusion” OR “transplantation” OR “implanted” OR “transplant” OR “implantation” OR “migration” OR “preservation” OR “grafting” OR “ischemia” OR “perfusion” OR “conservation” OR “preserve” OR “reservation”). Only original articles and reviews written in English and published between 2011 and 2021 were included. This query resulted in 429 records, which were obtained for this study.

### 2.2. Data collection and analysis

The methods of bibliometrics and scientific knowledge atlas are used to perform a visual analysis of the retrieved literature. All records retrieved from the WoSCC were downloaded independently by two authors (MC, QZ), and they included the number of annual publications; the output of countries/regions, institutions, journals, and authors; and citation frequency. Journal Citation Reports (JCR) 2020 was used to obtain the impact factor (IF). Any disagreements were resolved by consensus. Then, the data were converted to Microsoft Excel 2019 (Redmond, Washington, USA) and CiteSpace 5.8. R3 (Drexel University, Philadelphia, PA, USA) for the analysis of basic metrics. Microsoft Excel 2019 was applied to analyze and plot the annual publication output, total and mean IF, citations per article, and the total number of citations for every country/region and to organize data on the basic characteristics of publications and citations.

CiteSpace is a tool for analyzing scientific literature by creating visual models that represent fields of knowledge and their evolution over time. It also incorporates algorithms to analyze co-citation, collaboration, and hybrid networks in greater detail. Co-citation is when two related articles (or authors or journals) are simultaneously cited in a third article (or author or journal). Articles with a common topic tend to cluster around the same co-cited pair of articles, which helps identify the importance and relevance of the articles (24). This article focuses on co-author networks, co-occurring keywords, and co-cited references from CiteSpace. Therefore, research on H_2_S in the field of cardiac preservation can be visualized, which helps clearly explore the research status, development history, and future trends. We used CiteSpace to conduct a co-citation analysis of the authors, journals, references, and clusters, and further constructed a timeline view of co-cited references, by which we could clarify the rise and period of certain clustering fields. Furthermore, CiteSpace captures keywords with strong citation bursts and constructs visualization maps of all items. Yang et al. found that higher centrality has played a vital role in international cooperation, which is greatly influenced by the number of publications (25). A citation burst is a key indicator for identifying emerging trends. Reference bursts are references that have been cited in large numbers over time. Burst strength is an index used to measure citation intensity, and the greater the burst strength, the higher the citation intensity (26). The procedural steps required in CiteSpace are as follows: time slicing, thresholding, modeling, pruning, merging, and mapping (27). We set the “years per slice” and “top N per slice” values as 1 and 50, respectively; thus, the network map was extracted from the top 50 cited articles in 1 year per slice.

## 3. Results

### 3.1. Publication output and temporal trend

**Figure 1:**
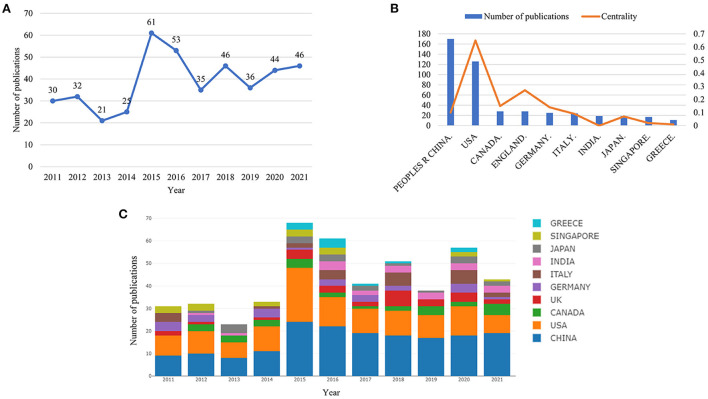
Trends in the number of publications and analysis of country/regions in hydrogen sulfide in heart preservation research. **(A)** The annual worldwide publication output. **(B)** Numbers, centrality for the top 10 countries/region. **(C)** Number of publications output from the top 10 countries.

A total of 429 publications met the inclusion criteria, comprising 314 articles and 115 reviews. The annual trend of H_2_S in the cardiac preservation research literature is shown in Figure 1A. It is found that the number of research articles in this field has shown a fluctuating upward trend in the last 10 years, roughly exhibiting a slow development stage (2011–2014), a rapid growth stage (2015–2016), and a plateau (2017–2021). From 2011 to 2014, the average annual publication volume of H_2_S in the field of cardiac preservation was relatively small, generally below 32, and even had a downward trend. In 2014, Polhemus et al. discovered that H_2_S plays a cellular protective role as an endogenous gas signaling molecule in cardiovascular diseases (28), which attracted a certain amount of attention from the academic community; in response, the number of publications began to grow. However, after 2017, the average annual publication volume declined and gradually stabilized.

### 3.2. Distribution by country/region and institution

**Figure 2:**
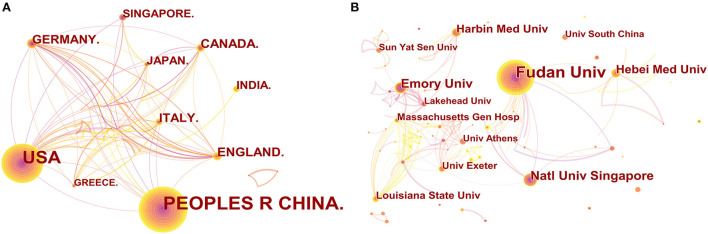
CiteSpace network visualization map of country/regions and institutions involved in hydrogen sulfide in heart preservation research. **(A)** Collaboration analysis of countries/regions. Of the 54 countries/ regions, 10 had at least 10 publications. **(B)** Collaboration analysis of institutions. Of the 272 institutions, 5 had at least 10 publications.

All publications were distributed among 54 countries/regions and 272 institutions (the number of nodes N represents the number of corresponding countries/regions and institutions. Figure 2A). The People's Republic of China had the highest level of production, with 170 documents, followed by the USA (126), Germany (25), and Italy (24), while Canada and the United Kingdom (28) shared the third place (Table 1). We further identified the annual national output of the 10 most productive countries/regions (Figure 1C). The figure shows that in the last 10 years, China has consistently been in the lead position in terms of publication volume, matched only by the United States, whose number of publications was on par with those of China in 2011, 2012, 2014, and 2015. Despite its leading number of annual publications, China has a relatively low betweenness centrality (0.1), which refers to the ability to act as an intermediary in the whole relational network, far behind that of the United States, which has a centrality of 0.65 and ranks first (Figure 1B, Table 1). The United Kingdom has a high centrality of 0.27, second only to the United States, meaning it may also play a key role in international cooperation. This shows that China's research influence in this field is not as strong as that of the United States and the United Kingdom, which may be due to the lack of high-quality research results. Nevertheless, Chinese scholars have the highest degree of research enthusiasm for this topic.

**Table 1:** Top 12 productive country/regions and institutions related to hydrogen sulfide in heart preservation research.

**Rank**	**Articles (N)**	**Centrality**	**Countries/** **Regions**	**Rank**	**Articles (N)**	**Centrality**	**Institutions**	**Location**
1	170	0.1	Peoples R China	1	41	0.11	Fudan Univ	Peoples R China
2	126	0.65	USA	2	16	0.01	Natl Univ Singapore	Singapore
3	28	0.15	Canada	2	16	0.09	Emory Univ	USA
3	28	0.27	England	4	13	0.04	Hebei Med Univ	Peoples R China
5	25	0.14	Germany	5	12	0.02	Harbin Med Univ	Peoples R China
6	24	0.09	Italy	6	9	0.05	Louisiana State Univ	USA
7	19	0	India	7	8	0.17	Univ Exeter	UK
7	19	0.07	Japan	7	8	0.14	Massachusetts Gen Hosp	USA
9	17	0.02	Singapore	9	7	0	Univ South China	Peoples R China
10	11	0.01	Greece	9	7	0.05	Lakehead Univ	Canada
11	9	0.02	Netherlands	9	7	0.03	Sun Yat Sen Univ	Peoples R China
12	8	0.01	Hungary	9	7	0	Univ Athens	Greece

To investigate international collaborations, we used CiteSpace to construct a network visualization map. Figure 2A shows collaborations among countries/regions publishing more than 10 documents (10 of 54). The number of posts is presented in the form of the size of nodes, the close cooperation between authors is presented in the form of thick and thin lines, and the dates of publications are presented in the form of cold and warm colors. As a country with the strongest betweenness centrality and the second largest number of publications, the United States collaborated the most with Sweden, Spain, Russia, Iran, and Switzerland.

To reveal collaborations between institutions, we used CiteSpace to analyze institutions of the extracted publications and generated a knowledge graph of the distribution of these institutions (Figure 2B). The top 12 institutions with relevant research results are shown in Table 1. Fudan University ranks first with the absolute leading number of articles (41, China), followed by the National University of Singapore (16, Singapore), Emory University (16, USA), Hebei Medical University (13, China), and Harbin Medical University (12, China), with a similar number of publications. Moreover, Fudan University cooperated closely with other organizations in China and the National University of Singapore. This indicates that Fudan University possibly has good research conditions in this field and exerts a strong influence in China; it also has a certain influence internationally. In addition, Figure 2B demonstrates that institutions in relevant research are concentrated mainly in universities and research institutes in China and the United States, and consequently, international cooperation needs to be strengthened.

### 3.3. Distribution by journal

A total of 429 publications on H_2_S in heart preservation research were published in 66 academic journals. The 13 most productive and co-cited journals are listed in Table 2. There was no significant difference in the number of articles published in these 13 journals. The journal *Oxidative Medicine and Cellular Longevity* published the most articles (14 publications, 3.263%), which had an IF of 6.543 in 2020 and only one more publication than *Antioxidants Redox Signaling* (13 publications, 3.030%). *Antioxidants Redox Signaling* has the highest IF of 8.401 among the 13 most productive journals, indicating that *Antioxidants Redox Signaling* made great contributions to this field.

**Table 2:** Top 13 productive journals of hydrogen sulfide in heart preservation research.

**Rank**	**Productive journal**	**Count (N)**	**Percentage (N/429)**	**IF (2020)**	**Rank**	**Co-cited journal**	**Count (N)**	**IF (2020)**
1	Oxid Med Cell Longev	14	3.263	6.543	1	Proc Natl Acad Sci USA	304	9.661
2	Antioxid Redox Signal	13	3.030	8.401	2	Circulation	297	29.69
3	Am J Physiol Heart Cir Physiol	12	2.797	4.733	3	Am J Physiol Heart Cir Physiol	285	-
3	Int J Mol Sci	12	2.797	5.923	4	Circ Res	275	17.367
5	Nitric Oxide	11	2.564	4.427	5	Antioxid Redox Signal	272	8.401
6	Life Sci	10	2.331	5.037	6	Biochem Bioph Res Co	237	3.575
6	Mol Med Rep	10	2.952	2.309	6	J Biol Chem	237	5.157
6	PLOS ONE	10	3.240	3.24	8	Cardiovasc Res	217	10.787
9	Int Mol Med	8	1.865	4.101	9	Brit J Pharmacol	205	-
10	Antioxidants	7	1.632	6.313	10	PLOS ONE	203	3.240
10	Front Physio	7	1.632	4.566	11	J Mol Cell Cardiol	198	5.000
10	J Am Heart Assoc	7	1.632	5.501	12	Faseb J	195	5.191
10	Pharmaco Res	7	1.632	7.658	13	Science	192	47.728

The most frequently co-cited journal was *P Natl Acad Sci USA* (304 citations), which had an IF of 9.661 in 2020. The next most frequently co-cited journals were *Circulation* (297 citations), *American Journal of Pgysiology-Heart and Circulation Physiology* (285 citations), *Circulation Research* (275 citations), and *Antioxidants Redox Signaling* (272 citations). It is worth noting that the IFs of the most frequently co-cited journals are generally higher than those of the most productive journals, which shows that scholars in related fields may tend to refer to some more authoritative journals. However, it cannot be ruled out that high citations may lead to high IFs.

### 3.4. Analysis of author and co-cited authors

**Figure 3:**
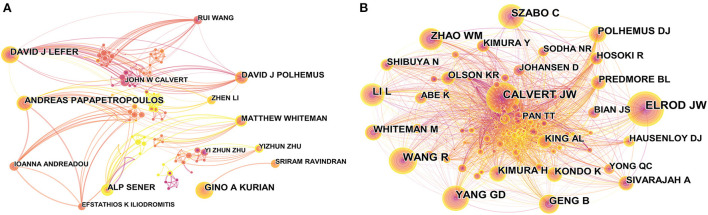
CiteSpace network visualization map of authors and co-cited authors of the articles related to hydrogen sulfide in heart preservation research. **(A)** CiteSpace network visualization map of authors. **(B)** CiteSpace network visualization map of co-cited authors.

CiteSpace was used to visually analyze the authors of the sample documents and generate a co-occurrence knowledge map of H_2_S researchers in the field of cardiac preservation (Figure 3A). A total of 343 authors contributed to all outputs of research in this area.

Table 3 lists the 10 most prolific authors in terms of the number of publications. A total of 5 authors have published more than 9 articles, and they are important core scholars in the study of H_2_S in cardiac preservation. Among them, David J Lefer and Gino A Kurian have published 12 articles, ranking first in the number of publications; they are the two most significant nodes in the graph. Second, Andreas Papapetropoulos (11 articles), Alp Sener, and David J Polhemus (9 articles) also have a high number of publications, forming important nodes in the graph.

**Table 3:** Top 12 productive authors and co-cited authors in hydrogen sulfide in heart preservation research.

**Rank**	**Author**	**Count**	**Centrality**	**Rank**	**Co-cited author**	**Citation**	**Centrality**
1	David J. Lefer	12	0.08	1	Elrod JW	173	0.01
1	Gino A. Kurian	12	0	2	Calvert JW	156	0.02
3	Andreas Papapetropoulos	11	0.02	3	Wang R	132	0.01
4	Alp Sener	9	0.02	4	Yang GD	128	0.04
4	David J. Polhemus	9	0.01	5	Szabo C	122	0.01
6	Matthew Whiteman	8	0.1	6	Li L	116	0.04
7	John W. Calvert	6	0.01	7	Zhao WM	115	0.07
7	Sriram Ravindran	6	0	8	Geng B	97	0.03
7	Zhen Li	6	0.01	9	Kimura H	87	0.03
7	Rui Wang	6	0.04	10	Polhemus DJ	83	0.03
7	Yizhun Zhu	6	0.06	11	Whiteman M	82	0.04
7	Ioanna Andreadou	6	0	12	Kondo K	76	0.05

The network visualization map of the co-cited authors is shown in Figure 3B. The largest nodes are associated with the most frequently co-cited authors, including Elrod JW (173 citations), Calvert JW (156 citations), Wang R (132 citations), Yang GD (128 citations), and Szabo C (122 citations) (Table 3). Of them, the top 12 highly productive authors do not overlap with the top 12 most frequently co-cited authors. This shows that the number of articles published by an author is not directly proportional to the number of citations, and the author with more articles published is not necessarily the author with the most citations.

### 3.5. Analysis of co-cited references

**Figure 4:**
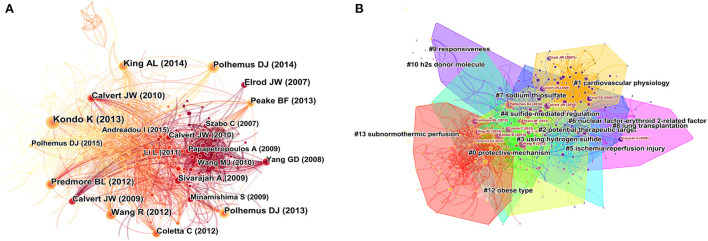
Analysis of references to hydrogen sulfide in heart preservation research. **(A)** Network map of co-cited references. **(B)** Network map of co-cited clusters.

To present more visual features of H_2_S in the field of cardiac preservation, we further used CiteSpace to construct a related reference co-citation network. The network map of co-cited references consisted of 580 references from the 50 most frequently cited references, with the time slice set as 1 year and the time-span set from 2011 to 2021 (Figure 4A). As shown in Table 4, the top 10 co-cited references were mainly published in *Circulation* (2 publications; IF 2020, 29.690), *Proc Natl Acad Sci USA* (2 publications; IF 2020, 11.205), *Circ Res* (2 publications; IF 2020, 17.367), *Am J Physiol Heart Circ Physiol* (2 publications; IF 2020, 4.733), *Circ Heart Fail* (1 publication; IF 2020, 8.790), and *Physiol Rev* (1 publication; IF 2020, 37.312). This indicates that the top 10 co-cited references with the highest frequency were published in high-quality journals.

**Table 4:** Top 10 co-cited references in hydrogen sulfide in heart preservation research.

**Rank**	**Co-citation counts**	**Centrality**	**Author**	**Year**	**Source**	**DOI**
1	57	0.08	Kondo K	2013	Circulation	doi: 10.1161/CIRCULATIONAHA.112.000855
2	43	0.06	King AL	2014	Proc Natl Acad Sci USA	doi: 10.1073/pnas.1321871111
3	38	0.09	Calvert JW	2010	Circulation	doi: 10.1161/CIRCULATIONAHA.109.920991
4	36	0.06	Polhemus DJ	2013	Circ Heart Fail	doi: 10.1161/CIRCHEARTFAILURE.113.000299
5	36	0.03	Polhemus DJ	2014	Circ Res	doi: 10.1161/CIRCRESAHA.114.300505
6	36	0	Elrod JW	2007	Proc Natl Acad Sci USA	doi: 10.1073/pnas.0705891104
7	36	0.05	Wang R	2012	Physiol Rev	doi: 10.1152/physrev.00017.2011
8	35	0.16	Predmore BL	2012	Am J Physiol Heart Circ Physiol	doi: 10.1152/ajpheart.00044.2012
9	30	0.1	Peake BF	2013	Am J Physiol Heart Circ Physiol	doi: 10.1152/ajpheart.00796.2012
10	29	0.04	Calvert JW	2009	Circ Res	doi: 10.1161/CIRCRESAHA.109.199919

In terms of the centrality scores (Table 4), the most prominent article in visualization is the one written by Predmore in 2012 (Centrality = 0.16) (29). Predmore's study showed that *in vivo* diallyl trisulfide (DATS) administration resulted in the prolonged release of low H_2_S, protecting ischemic myocardium through multiple mechanisms, speculating that DATS therapy may be an attractive option for the treatment of acute myocardial infarction. The publications Bibli (2015; Centrality = 0.14) (30) and Peake (2013; Centrality = 0.10) (31) follow it. These prominent publications separately demonstrate the protective effects of endogenous H_2_S on the heart through different mechanisms, such as the cGMP/PKG/PLN pathway. The size of these notable nodes indicates that they are well-cited and can serve as a more important reference for H_2_S in the field of cardiac preservation.

**Figure 5:**
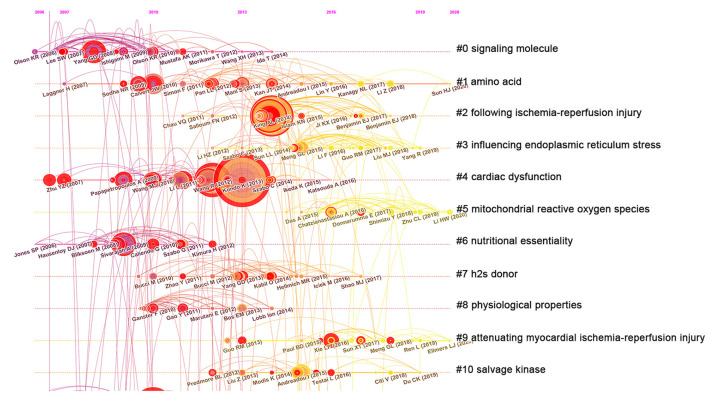
Timeline view of co-cited references related to hydrogen sulfide in heart preservation research.

Cluster #0 (signaling molecule) has the darkest and most nodes but does not contain the 10 longest-cited references and only contains references from before 2014. Cluster #5 (mitochondrial reactive oxygen species) has the brightest color and contains 35 references, indicating it is the most recently formed cluster and the most popular research hotspot and direction. Cluster #2 (following ischemia–reperfusion injury) and cluster #4 (cardiac dysfunction) have the largest nodes scattered across the timeline and contain 7 of the 10 most frequently cited references, including Kondo K (2013), King AL (2014), and Wang R (2012) (Figures 4B, 5). Therefore, future research should focus on the application of H_2_S in heart failure; in addition, studies have shown that reducing reperfusion injury during myocardial ischemia can maintain the vitality of the heart, and more high-quality basic research is needed to further clarify potential related mechanisms.

### 3.6. Analysis of co-occurring keywords

**Figure 6:**
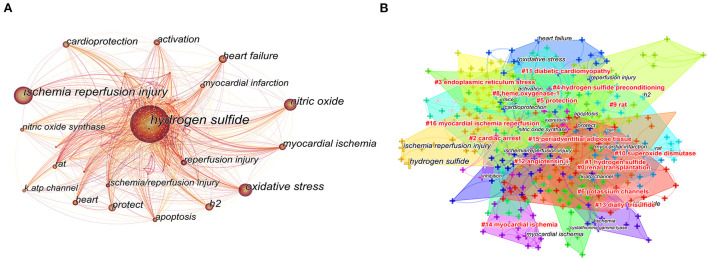
Analysis of keyword co-occurrence **(A)** CiteSpace network visualization map of co-occurring keywords **(B)** CiteSpace network visualization map of co-occurring keyword clusters.

Keywords reflect the concentration and refinement of the core content of the literature, and keywords that appear frequently may indicate the research hotspots in this field. Therefore, by using the keyword visualization analysis function of CiteSpace, the knowledge map of keyword co-occurrence in H_2_S research in cardiac preservation was drawn to grasp the main research hotspots in this field (Figure 6A). As shown in Figure 6A, “hydrogen sulfide,” “ischemia reperfusion injury,” and “oxidative stress” had the highest frequencies (277, 143, and 102, respectively), becoming the three core nodes in the graph. At the same time, “nitric oxide,” “heart failure,” “h2,” and “myocardial ischemia” all appeared more than 60 times. If the qualifiers of the study area, such as H_2_S, are excluded and combined with the statistical results of other main keywords in the map, the main research contents of H_2_S in the field of cardiac preservation focus on “ischemia reperfusion injury” and “oxidative stress.” Among them, “ischemia reperfusion injury” has received the most attention and is the core research topic in this field. However, not all high-frequency keywords have high centrality. The centrality of myocardial ischemia was 0.14, which to some extent represents the focus of this study. Nitric oxide was 0.13, second only to myocardial ischemia (Table 5).

**Table 5:** Top 12 keywords in hydrogen sulfide in heart preservation research.

**Rank**	**Count**	**Centrality**	**Keywords**
1	277	0.02	Hydrogen sulfide
2	143	0.08	Ischemia reperfusion injury
3	102	0.09	Oxidative stress
4	97	0.13	Nitric oxide
5	69	0.07	Heart failure
6	68	0.05	H_2_
7	62	0.14	Myocardial ischemia
8	59	0.06	Protect
9	57	0.07	Activation
10	56	0.06	Cardioprotection
11	51	0.08	Heart
12	50	0.08	Reperfusion injury

At the same time, we used CiteSpace to create a knowledge map of keyword co-occurrence clusters after combining synonyms into 16 clusters (Figure 6B), including “renal transplantation,” “hydrogen sulfide,” “reperfusion injury,” and “hydrogen sulfide preconditioning.” Cluster #0 (renal transplantation) has the deepest color and the most nodes, but it is not included in the top 10 most cited keywords, indicating that H_2_S was applied to renal transplantation in the early stage, but it is not the current research focus and direction. Cluster #3 (endoplasmic reticulum stress) has the brightest color and contains 26 keywords, indicating it is the most recently formed cluster and the hottest research hotspot and direction at present.

**Figure 7:**
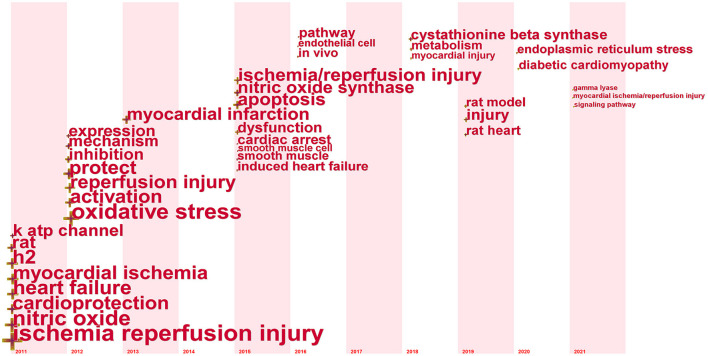
CiteSpace overlay visualization of the keyword evolvement by time.

Figure 7 shows a visualization of the keyword evolution over time, and the keywords that appear in each time zone are the words that appear for the first time in the time range of 2011–2021. During the early stage of research on “ischemia reperfusion injury,” “nitric oxide,” “heart failure,” “myocardial ischemia,” and “cardio protection” were the major topics in this field in 2011. During the latest stage of research on “ischemia reperfusion injury,” “signaling pathway,” “myocardial ischemia/reperfusion injury,” and “gamma lyase,” were the major topics in this field in 2021. The keyword ischemia reperfusion injury was used throughout the study period. This indicates that current research focuses on the signaling pathway mechanism of H_2_S on cardiac protection and the study of related enzymes.

### 3.7. Analysis of keyword bursts

**Figure 8:**
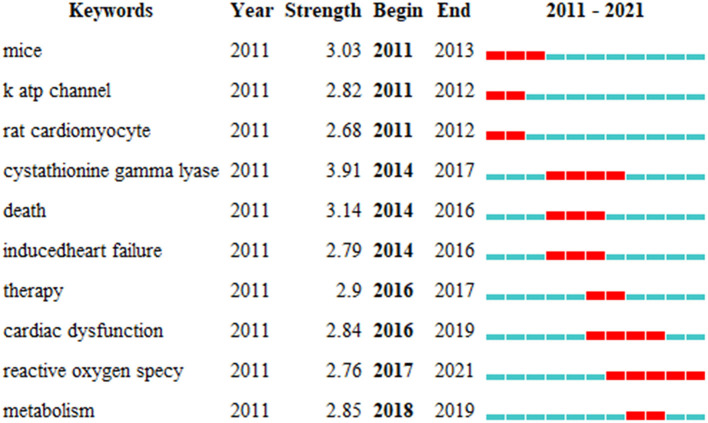
Keywords with periods of burst from 2011 onward are among the top 10 burst keywords in articles related to hydrogen sulfide in heart preservation research.

We used CiteSpace to detect keyword bursts to determine the hotspots and research frontiers over time. Keyword bursts from 2011 onward are among the top 10 keyword bursts in articles related to H_2_S in heart preservation research (Figure 8). The keyword with the strongest burst was “cystathionine gamma lyase” (with a burst strength of 3.91), and the burst lasted 3 years (2014–2016). It is related to the mechanism by which H_2_S exerts cardioprotective effects and has attracted great attention. The word burst that lasted the longest was “reactive oxygen specy”; the burst started in 2017 and continues to present, with a strength of 2.76. Therefore, “reactive oxygen specy” is not only a long-lasting research hotspot but also the latest research hotspot, which deserves attention. Other listed keywords also have a burst strength >2, but their popularity declined as research progressed and new hotspots emerged.

## 4. Discussion

### 4.1. General information analysis

According to data from the Science Citation Index-Expanded database of the WoSCC database from 2011 to 2021, a total of 429 articles on the application of H_2_S in heart preservation were published in 66 journals by 343 authors from 54 countries and 272 institutions.

In recent years, the application of H_2_S in heart preservation has received increasing attention from experts. In 2004, Geng et al. found for the first time that H_2_S can effectively protect myocardial contractile activity by directly scavenging oxygen-free radicals and reducing the accumulation of lipid peroxidation (32). Then, Johansen et al. (33) found that H_2_S prevented myocardial ischemia–reperfusion injury, and K-ATP was involved (33). In the same year, Bian et al. and Sivarajah et al. used mouse models to explore the mechanism of H_2_S in cardiac protection, which opened a new door for applying H_2_S in the heart (34, 35). We searched for the application of H_2_S in heart preservation research from 2011 to 2021. Until 2013, the number of annual publications maintained a steady low level. After 2013, the annual publication production steadily increased, peaking in 2015 (Figure 1A). This trend is associated with the research on H_2_S protecting against pressure overload-induced heart failure *via* upregulation of endothelial nitric oxide synthase basic experiment that was published in 2013, with the highest citation frequency (36). This research is considered the most basic and essential, which has extensively promoted the development of this field of study.

In the country/region analysis, the high centrality (≥0.10) node indicates the “bridge” effect of these countries/regions in the global cooperation network. Of the top 12 institutions with the most publications, five are from China, three are from the United States, one is from Canada, one is from the United Kingdom, one is from Germany, and one is from Singapore (Table 1). In addition, the centrality of the United States, the United Kingdom, Canada, Germany, and China is more significant than 0.1, indicating that these countries play an essential role in the global research networks on H_2_S in heart reservation. China (centrality = 0.1) published the greatest number of documents, with 173 articles, but the quality of the research is lower than that of the United States (centrality = 0.65). However, among the institutions for the study of H_2_S productive institutions, five Chinese institutions are among the top 12 productive institutions. Fudan University is at the top of the list, representing the significant participation of Chinese researchers.

In the journal analysis (Table 2), *PLOS ONE, Am J Physiol Heart Cir Physiol*, and *Antioxidants Redox Signaling* were found to be the three most significant, ranking among the top 13 in both productive and co-cited journals. This demonstrates that they were key sources for investigators in the field of medical hydrogen research. *Proc Natl Acad Sci USA* has the highest co-citations, partly because four of the seven highly cited articles were published in this journal (Table 4) (37, 38).

Among the top 12 productive authors and co-cited authors (Table 3), the top two most effective authors are David J Lefer (12 documents) and Gino A Kurian (12 documents). However, in terms of centrality, these authors are far lower than the author Matthew Whiteman, which indicates that although Whiteman does not have the largest number of publications, the quality of his publications is high. He has a strong influence on international cooperation. As shown in Figure 3A, he has recently worked in close cooperation with Alp Sener. Among the top 10 co-cited authors, the articles published by Elrod JW, Calvert JW, Polhemus DJ, and Kondo K are among the top 10 most cited (Tables 3, 4) (19, 28, 36, 37, 39).

### 4.2. Reference analysis

Of the top 10 co-cited articles, only two were reviews, while eight were mechanistic investigations of H_2_S in protecting cardiac function; moreover, these studies have been regarded as reliable reference resources for subsequent related studies. Notably, the animal experiments by Kondo (36) in 2013, the most influential article, suggest that the maintenance of endogenous H_2_S levels can play an important role in maintaining cardiac function during the development of heart failure and preventing damage from compensation. Moreover, oral H_2_S therapy can prevent the transition from decompensated cardiac hypertrophy to decompensated cardiac hypertrophy. Therefore, H_2_S therapy may become a novel and promising treatment for left ventricular dysfunction. In addition, King et al. used a variety of H_2_S-releasing compounds to confirm that the protective effects of H_2_S on cells are not limited to specific H_2_S donors. Among them, CSE KO mice showed reduced NO levels and reduced NO synthesis by endothelial NO synthase (eNOS), i.e., the cytoprotective effect elicited by CSE-derived H_2_S was eNOS/NO-dependent. Both oxidative stress and myocardial injury in mice were attenuated by exogenous H_2_S treatment, so H_2_S may have clinical applications (38). In 2010, Calvert et al. found that either using exogenous H_2_S or modulating H_2_S production could play an important role in the treatment of ischemic heart failure (40). Furthermore, experimental results by Polhemus in 2013 showed that H_2_S promotes the growth of new blood vessels by creating a proangiogenic environment, ultimately reducing ventricular remodeling and improving cardiac function in patients with heart failure (39).

Furthermore, experimental results by Polhemus in 2013 showed that H_2_S promotes the growth of new blood vessels by creating a proangiogenic environment, ultimately reducing ventricular remodeling and improving cardiac function in patients with heart failure (39). In addition, there are five American references in the top 10. Therefore, the United States has the best academic reputation for the study of H_2_S, which is consistent with the centrality of Table 1.

In addition, according to Figure 5, Cluster #0 (signaling molecule) does not contain relevant references after 2014, indicating that it is not a current hot topic. The main reason may be that scholars' research on signaling molecules has reached a significant level and does not require further investigation. It is worth noting that Cluster #5 (mitochondrial reactive oxygen species) and Cluster #9 (attenuating myocardial ischemia–reperfusion injury) continue to the present, indicating that they are the current hotspots of H_2_S in heart preservation. Some studies have shown that H_2_S can protect the myocardium by inhibiting mitochondrial complex IV and reducing the level of ROS in cardiomyocytes (41). mTORC2 phosphorylation of Akt is a crucial mediator of H_2_S-induced cardioprotection in I/R (42, 43). Therefore, future studies on the role of H_2_S in cardiac preservation tend to target H_2_S delivery to mitochondria to reduce reactive oxygen species produced during cardiac ischemia–reperfusion injury, thereby achieving cardiac conservation.

### 4.3. Keywords analysis

According to Figure 6B, Cluster #11 (diabetic cardiomyopathy) suggests that H_2_S may also be essential in treating diabetes-related cardiovascular complications. Some studies have found that H_2_S can reduce cardiac dysfunction induced by a high-fat diet by inhibiting ER stress (44). Keyword co-occurrence analysis and burst keywords reflected the developing trends and hotspots of sulfide in cardiac preservation (Figures 6, 8). Hotspot research on mitochondrial reactive oxygen species are consistent with reference analysis. It is worth noting that nitric oxide (centrality = 0.13) was included in the top 10 keywords, and centrality was second only to myocardial ischemia (centrality = 0.14), possibly because the cytoprotective signaling of H2S-mediated ischemia–reperfusion injury largely depends on nitric oxide production (45).

## 5. Conclusion

This study is the first bibliometric analysis of the application of H_2_S in cardiac preservation. Especially since 2013, an increasing number of studies have confirmed the medical value of H_2_S in heart-related diseases. China and the United States have made the most significant contributions in this area. Fudan University was the most productive institution. *Oxidative Medicine and Cellular Longevity* were the journals with the greatest number of publications. David J. Lefer and Gino A. Kurian were the most influential authors. Most studies have focused on mouse models of the protective effects of H_2_S on cardiomyocytes.

Regarding the role of H_2_S in cardiac preservation, future research hotspots tend to investigate targeting the mitochondria to reduce reactive oxygen species produced during myocardial ischemia–reperfusion injury. Based on this, further research on other molecular mechanisms of H_2_S protection in the heart will provide therapeutic targets and new clinical treatment ideas for cardiovascular diseases.

## Data availability statement

The original contributions presented in the study are included in the article/supplementary material, further inquiries can be directed to the corresponding author.

## Author contributions

MC conceived the study, contributed part of the figures, and revised the manuscript for important intellectual content. QZ and FW performed the literature search and part of the figures. FS, YM, YZ, and MZ edited the manuscript. MZ provided financial support. All authors contributed to the article and approved the submitted version.
